# Oligodendrocyte-Specific Deletion of *FGFR1* Reduces Cerebellar Inflammation and Neurodegeneration in MOG_35-55_-Induced EAE

**DOI:** 10.3390/ijms22179495

**Published:** 2021-08-31

**Authors:** Ranjithkumar Rajendran, Vinothkumar Rajendran, Mario Giraldo-Velasquez, Fevronia-Foivi Megalofonou, Fynn Gurski, Christine Stadelmann, Srikanth Karnati, Martin Berghoff

**Affiliations:** 1 Experimental Neurology, Department of Neurology, University of Giessen, Klinikstrasse 33, 35385 Giessen, Germany; vinothkumar.rajendran@neuro.med.uni-giessen.de (V.R.); Fevronia.F.Megalofonou@med.uni-giessen.de (F.-F.M.); fynn.gurski@med.uni-giessen.de (F.G.); martin.berghoff@neuro.med.uni-giessen.de (M.B.); 2 Department of Neurology, Sozialstiftung Bamberg, Buger Strasse 80, 96049 Bamberg, Germany; Mario.Giraldo@sozialstiftung-bamberg.de; 3 Institute of Neuropathology, University Medical Center Göttingen, Robert-Koch-Strasse 40, 37099 Göttingen, Germany; cstadelmann@med.uni-goettingen.de; 4 Institute of Anatomy and Cell Biology, University of Würzburg, Koellikerstrasse 6, 97080 Würzburg, Germany; srikanth.karnati@uni-wuerzburg.de

**Keywords:** FGFR1, oligodendrocytes, demyelination, inflammation, cerebellum, EAE, MS

## Abstract

Multiple sclerosis (MS) is a chronic inflammatory and degenerative disease of the central nervous system (CNS). MS commonly affects the cerebellum causing acute and chronic symptoms. Cerebellar signs significantly contribute to clinical disability, and symptoms such as tremor, ataxia, and dysarthria are difficult to treat. Fibroblast growth factors (FGFs) and their receptors (FGFRs) are involved in demyelinating pathologies such as MS. In autopsy tissue from patients with MS, increased expression of FGF1, FGF2, FGF9, and FGFR1 was found in lesion areas. Recent research using mouse models has focused on regions such as the spinal cord, and data on the expression of FGF/FGFR in the cerebellum are not available. In recent EAE studies, we detected that oligodendrocyte-specific deletion of *FGFRs* results in a milder disease course, less cellular infiltrates, and reduced neurodegeneration in the spinal cord. The objective of this study was to characterize the role of *FGFR1* in oligodendrocytes in the cerebellum. Conditional deletion of *FGFR1* in oligodendrocytes (*Fgfr1^ind−/−^*) was achieved by tamoxifen application, EAE was induced using the MOG_35-55_ peptide. The cerebellum was analyzed by histology, immunohistochemistry, and western blot. At day 62 p.i., *Fgfr1^ind−/−^* mice showed less myelin and axonal degeneration compared to FGFR1-competent mice. Infiltration of CD3(+) T cells, Mac3(+) cells, B220(+) B cells and IgG(+) plasma cells in cerebellar white matter lesions (WML) was less in *Fgfr1^ind−/−^*mice. There were no effects on the number of OPC or mature oligodendrocytes in white matter lesion (WML). Expression of FGF2 and FGF9 associated with less myelin and axonal degeneration, and of the pro-inflammatory cytokines IL-1β, IL-6, and CD200 was downregulated in *Fgfr1^ind−/−^* mice. The FGF/FGFR signaling protein pAkt, BDNF, and TrkB were increased in *Fgfr1^ind−/−^* mice. These data suggest that cell-specific deletion of *FGFR1* in oligodendrocytes has anti-inflammatory and neuroprotective effects in the cerebellum in the EAE disease model of MS.

## 1. Introduction

Multiple sclerosis (MS) is a chronic inflammatory and degenerative disease of the central nervous system (CNS) associated with oligodendrocyte injury, demyelination, and degeneration of axons [1]. As a consequence of demyelination, oligodendrocytes differentiate, proliferate, and migrate to repair damaged myelin sheaths [2,3,4]. This repair mechanism is effective in early stages of MS, but it deteriorates with disease progression [5,6,7,8,9]. The underlying mechanisms of impaired remyelination include failure of oligodendrocyte progenitor cell (OPC) recruitment and differentiation of OPCs into mature oligodendrocytes [8,10], oxidative stress, and mitochondrial dysfunction [11].

FGF/FGFR signaling pathways are involved in the pathogenesis of MS and its disease models. In autopsy tissue, an increased number of FGF1/2+ macrophages and astrocytes were observed in and around active lesions in the cerebral cortex and periventricular white matter; additionally, FGFR1 was upregulated in oligodendrocyte progenitor cells (OPC) in active lesions and around chronic lesions [12,13]. Analysis of the cerebrospinal fluid (CSF) of patients with relapsing-remitting MS showed higher levels of FGF2 expression than healthy controls and revealed that an increased concentration of FGF2 positively correlates with lesion load of disease activity [14]. Human data indicate the implication of FGF2 in the pathogenesis of MS. We have investigated oligodendrocyte-specific deletion of *FGFR* in experimental autoimmune encephalomyelitis (EAE) [15,16]. *Fgfr1^ind−/−^* and *Fgfr2^ind−/−^* mice showed less motor deficits, reduced inflammation, and demyelination in the spinal cord. Phosphorylation of Akt and ERK was regulated in the spinal cord of both *Fgfr1^ind−/−^* and *Fgfr2^ind−/−^* mice [15,16]. Consequently, data from this disease model of MS suggest that FGFR in oligodendrocytes exert negative effects.

The cerebellum plays a crucial role in coordination and balance [17]. In MS studies one third of subjects have functionally relevant cerebellar deficits [18,19]. Disabling symptoms such as nystagmus, ataxia, dysphagia, or dysarthria are caused by destruction within the cerebellum [20,21]. In fact, the prognosis is worse when patients have early signs of cerebellar deficits [22,23], and symptoms caused by lesions in the cerebellum respond poorly to symptomatic therapies [24,25]. Impairment of cerebellar function may also lead to a lower quality of life [26]. Thus, cerebellar dysfunction in MS has clinically relevant consequences, its underlying mechanism and treatment strategies remains poorly understood. Inflammation, demyelination, and axonal damage can be found in the whole CNS of patients with MS [17]; however, comparative data on the extent of damage in various regions of the CNS are limited. The number of lesions in the cerebellar white matter is lower than the number in the forebrain white matter. Further, axonal loss or remyelination differs between cerebellar and forebrain plaques, the underlying mechanisms are unclear [25]. Magnetic resonance imaging studies have confirmed extensive cerebellar involvement in both relapsing-remitting and progressive MS. Persistent cerebellar dysfunction has been linked with axonal loss in the cerebellum of patients with progressive MS [27].

In EAE, inflammation, demyelination, or neurodegeneration have been found in various regions of the CNS including the cerebrum, corpus callous, hippocampus, and the cerebellum [28]. Several studies have focused on the pathology of cerebellar structures in EAE [29,30,31,32]. In MOG_35-55_-induced EAE, demyelinating lesions and infiltrates of T cells and macrophages were detected in the cerebellum [28]. A reduced number of CD4 and CD8 positive T cells was found in the cerebellum of mice treated with an anti-CX3CL1 antibody [33]. Multilocular neuroinflammation and demyelination was found in the spinal cord and the cerebellum of astrocyte specific IL-23 producing mice [31]. Increased neutrophil infiltrations and demyelination were found in the cerebellum of Socs3 deficient mice [34], and hypoxia and increased variations in oxygenation were found in the grey matter of the cortex and the cerebellum [35]. In another study using MRI, a significant reduction in cerebellar and cerebral cortex volumes was detected [36].

Based on data from previous EAE studies, we hypothesized that oligodendrocyte-specific deletion of *FGFR1* results in reduced inflammation and degeneration in the cerebellum. In agreement with our hypothesis, we found reduced inflammatory infiltrates as well as less myelin and axonal degeneration in the cerebellum of *Fgfr1^ind−/−^* mice. Akt phosphorylation, TrkB, and BDNF expression were upregulated in *Fgfr1^ind−/−^* mice. In addition, we found reduced IL-6, IL-1β, and CD200 expression in *Fgfr1^ind−/−^* mice. The expression of FGF2 and FGF9 was reduced, and myelin protein PLP expression was increased. These data suggest that FGF/FGFR pathways are associated with proinflammatory mechanisms and myelination, and they may contribute to disability of EAE caused by cerebellar damage.

## 2. Results

### 2.1. Reduced Inflammatory Infiltrates in WML of the Cerebellum in Fgfr1^ind−/−^ Mice

**Figure 1:**
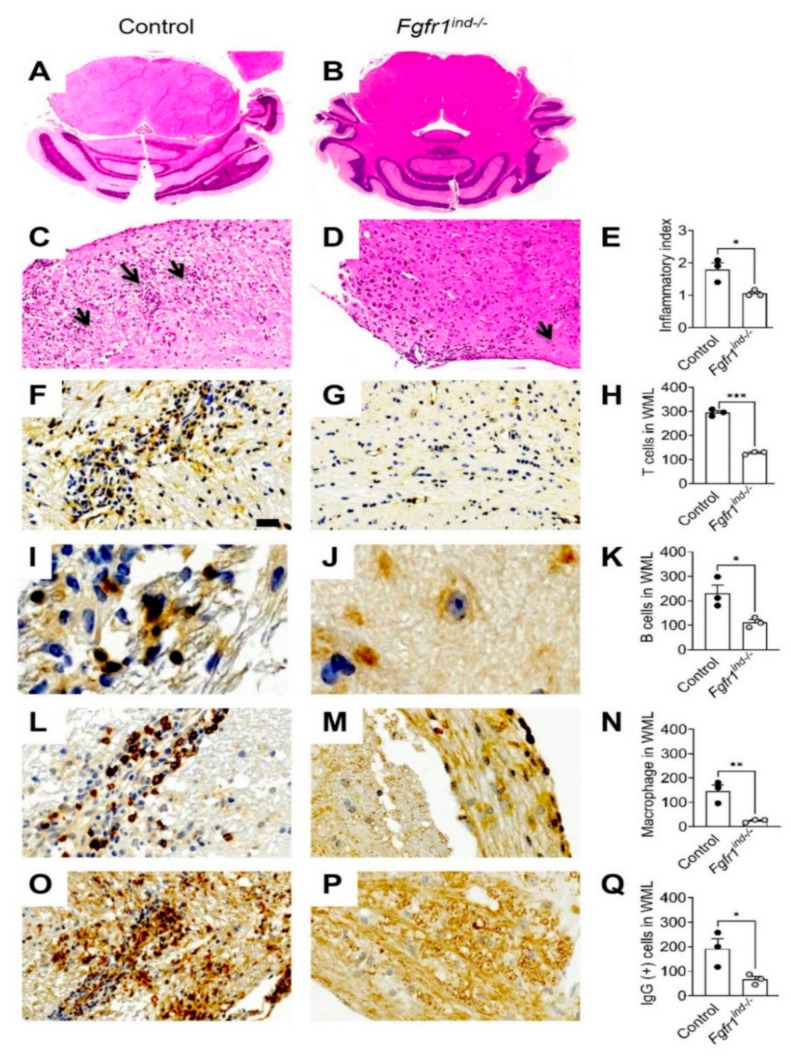
Inflammation and immune cell infiltration in the cerebellum at day 62 p.i. Cross section of the control and *Fgfr1^ind−/−^* mice cerebellum (**A**,**B**), and inflammation was shown as inflammatory index (**C**–**E**). Inflammation was less in *Fgfr1^ind−/−^* mice cerebellum (**C**–**E**). Arrowheads indicate immune cell clustering (**C**–**E**). Immune cell infiltration in cerebellar WML at day 62 p.i. *Fgfr1^ind−/−^* mice showed a decreased number of CD3(+) T cells (**F**–**H**), B220(+) B cells (**I**–**K**), MAC3(+) macrophages/microglia activation (**L**–**N**), and reduced IgG(+) plasma cell infiltration (**O**–**Q**) in WML of the cerebellum. *n* = 3/group. Scale bar depicts 50 μm (**F**–**P**). Data are expressed as the mean ± SEM. * *p* < 0.05, ** *p* < 0.01. *** *p* < 0.001.

To assess the effects of oligodendrocyte-specific deletion of *FGFR1* in the spinal cord, we previously reported a reduction in CD3(+) T cells, B220(+) B cells and Mac3(+) macrophages/activated microglia in *Fgfr1^ind−/−^* mice on day 62 p.i. [16]. In the cerebellum, *Fgfr1^ind−/−^* mice showed less inflammatory infiltrates at day 62 p.i. (*p* = 0.0251; Figure 1C–E). To study the composition of these infiltrates in WML of the cerebellum, we analysed different immune cell populations. CD3(+) T cells (*p* < 0.0001), B220(+) B cells (*p* = 0.0321), Mac3(+) activated microglia/macrophages (*p* = 0.0092) and IgG(+) plasma cells (*p* = 0.0428) were decreased in *Fgfr1^ind−/−^* mice (Figure 1F–Q).

### 2.2. Oligodendroglia-Specific Deletion of FGFR1 Is Associated with a Reduction in Inflammatory Mediators in the Cerebellum

**Figure 2:**
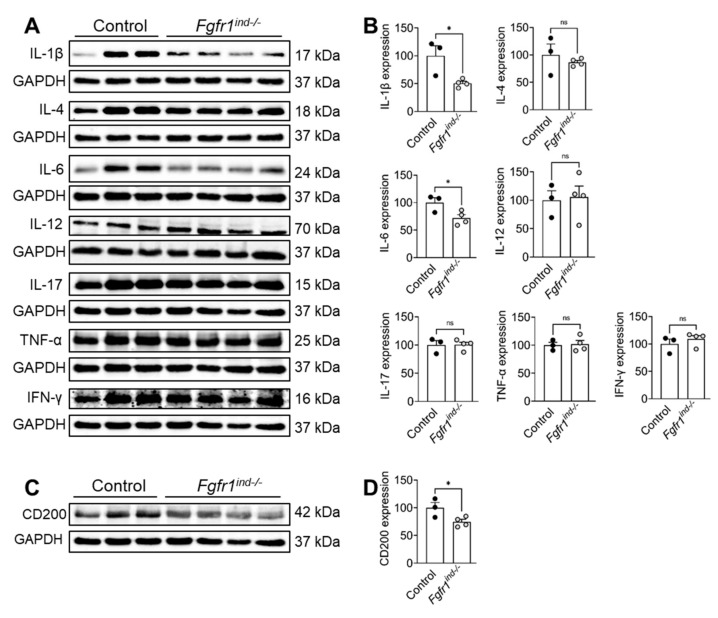
Protein expression of pro- and anti-inflammatory cytokines and CD200 in cerebellar lysates at day 62 p.i. (**A**,**B**) IL-1β and IL-6 expression were less in *Fgfr1^ind−/−^* mice. IL-4, IL-12, IL-17, TNF-α, and IFNγ expressions were not different between controls and *Fgfr1^ind−/−^* mice. (**C**,**D**) CD200 expression was reduced in *Fgfr1^ind−/−^* mice. *n* = 3–4/group. Data are expressed as the mean ± SEM. ns = not significant, * *p* < 0.05.

To investigate the effect of oligodendroglia-specific deletion of *FGFR1* on cytokine regulation in the cerebellum, whole cerebellar lysates were analysed by western blot. The proinflammatory cytokines IL-1β (*p* = 0.0254) and IL-6 (*p* = 0.0438) were downregulated in *Fgfr1^ind−/−^* mice (Figure 2A,B). In contrast, IL-12 (*p* = 0.834), IL-17 (*p* = 0.9600), TNFα (*p* = 0.8400) and IFNγ (*p* = 0.4240) were not regulated (Figure 2A,B). CD200 was expressed less in *Fgfr1^ind−/−^* mice (*p* = 0.0463, Figure 2C,D). Phosphorylation of JNK (*p* = 0.809) and P38 (*p* = 0.312) were not regulated in the cerebellum by the deletion of *FGFR1*.

### 2.3. Oligodendroglia-Specific Deletion of FGFR1 Reduces Myelin Loss and Axonal Damage in the Cerebellum

**Figure 3:**
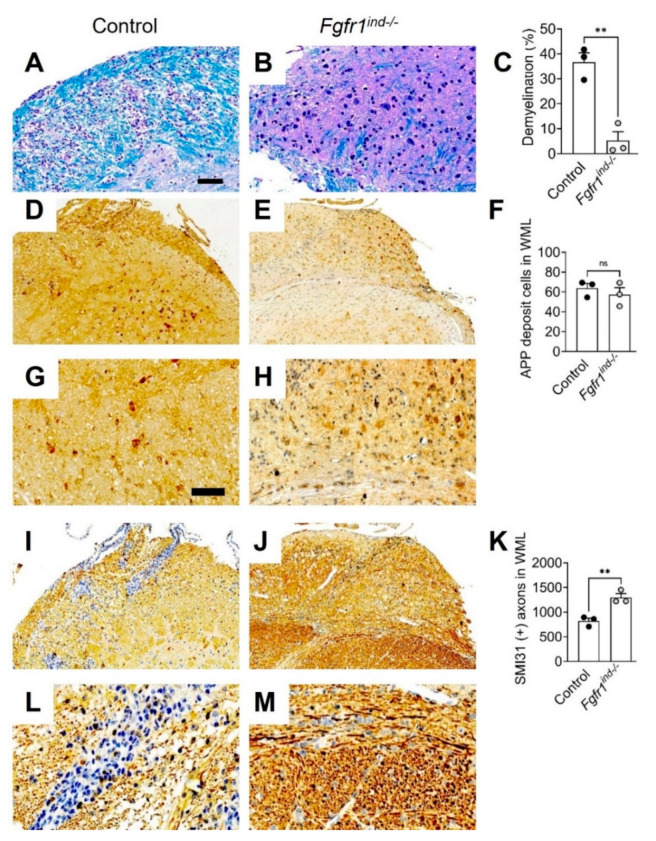
Demyelination (**A**–**C**) in the cerebellum at day 62 p.i. Demyelination was reduced in *Fgfr1^ind−/−^* mice (**A**–**C**). Quantification of the number of amyloid precursor protein (APP) (+) and SMI31(+) phosphorylated axons at day 62 p.i. The number of APP (+) axons was not different between controls and *Fgfr1^ind−/−^* mice (**D**–**F**). The number of SMI31(+) phosphorylated axons were higher in *Fgfr1^ind−/−^* mice (**I**–**K**). The dark yellow or brown staining was counted as APP and SMI31 positive cells. (**G**,**H**,**L**,**M**) were higher magnification of (**D**,**E,I**,**J**). Scale bars depicts 100 μm (**A**,**B**,**D**,**E**,**I**,**J**), scale bar depicts 50 μm (**G**,**H**,**L**,**M**). *n* = 3/group. Data are expressed as the mean ± SEM., ** *p* < 0.01.

In our recent study *Fgfr1^ind−/−^* mice showed less myelin and axon degeneration in the spinal cord at day 62 p.i. [16]. In the cerebellum, myelin loss (*p* = 0.0034; Figure 3A–C) was reduced in *Fgfr1^ind−/−^* mice at day 62 p.i. Although the number of APP(+) axons in WML was not altered at day 62 p.i. (*p* = 0.4857) (Figure 3D–F; G,H higher magnification), the number of SMI31(+) axons in WML was higher in *Fgfr1^ind−/−^* mice than controls (*p* = 0.0077) (Figure 3I–K; L,M higher magnification).

### 2.4. Number of Oligodendrocytes in WML and Myelin Expression in the Cerebellum of Fgfr1^ind−/−^ Mice

**Figure 4:**
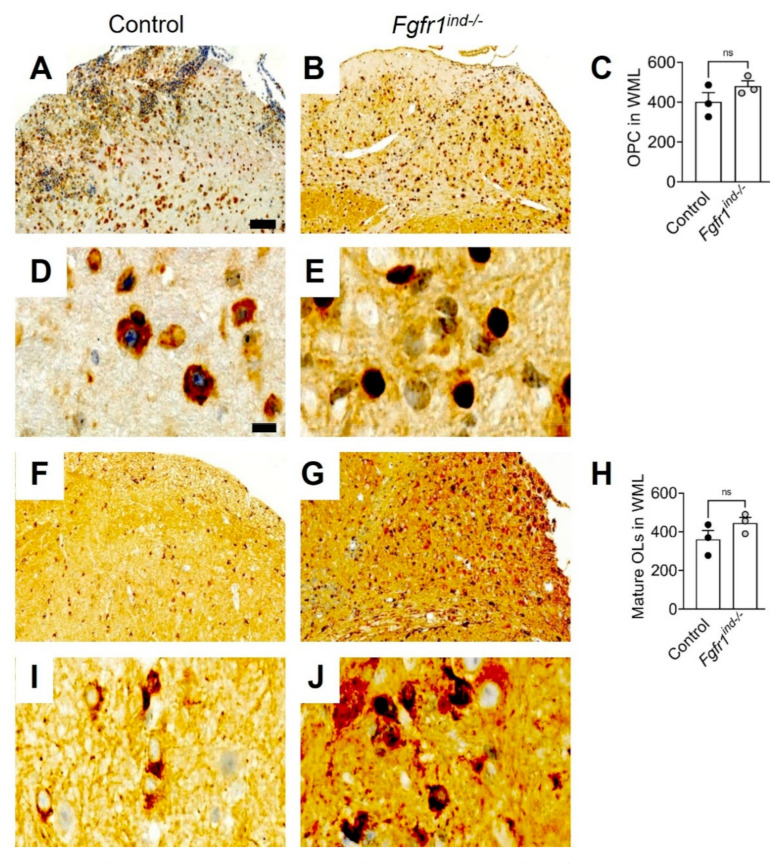
Oligodendrocyte populations at day 62 p.i. The number of Olig2(+) and NogoA(+) oligodendrocytes in WML of the cerebellum for controls (**A**,**D**,**F**,**I**) and *Fgfr1^ind−/−^* mice (**B**,**E**,**G**,**J**). There were no differences in Olig2(+) (**A**–**E**) and NogoA(+) (**F**–**H**) oligodendrocyte numbers between controls and *Fgfr1^ind−/−^* mice. (**D**,**E**,**I**,**J**) were magnified views of (**A**,**B**,**F**,**G**). *n* = 3/group. Scale bar depicts 50 μm (**C**,**D**). Data are expressed as the mean ± SEM. ns = not significant.

**Figure 5:**
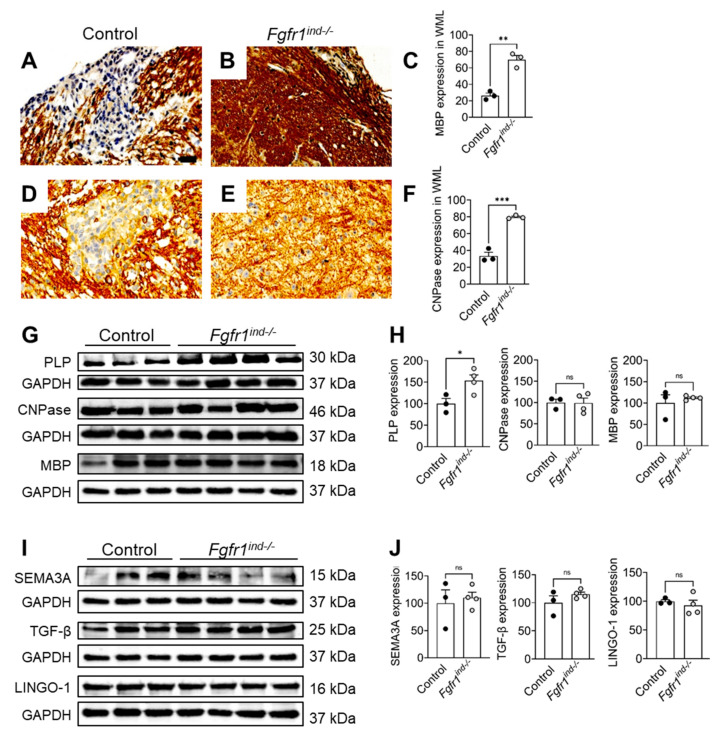
Myelin protein and myelin inhibitor expression at day 62 p.i. Increased MBP(+) (**A**–**C**) and CNPase(+) cells (**D**–**F**) in WML of the cerebellums of *Fgfr1^ind−/−^* mice. Myelin protein PLP was upregulated in *Fgfr1^ind−/−^* mice (**G**,**H**). Myelin proteins CNPase and MBP (**G**,**H**), and myelin inhibitors SEMA3A, TGF-β, LINGO-1 (**I**,**J**) were not regulated. Cerebellar tissue immunostaining: *n* = 3/group, cerebellar tissue lysate western blot: *n* = 3–4/group. Data are expressed as the mean ± SEM. ns = not significant, * *p* < 0.05, ** *p* < 0.01, *** *p* < 0.001.

We previously reported there were no alterations in the Olig2(+) (OPCs) and NogoA(+) (mature oligodendrocytes) oligodendroglial populations in spinal cord WML of *Fgfr1^ind−/−^* mice [16]. In the cerebellum, *FGFR1* deletion did not change the number of olig2(+) (*p* = 0.2125, Figure 4A–E) or NogoA(+) (*p* = 0.1998, Figure 4F–J) oligodendrocytes in WML. Expression of MBP (*p* = 0.0018, Figure 5A–C) and CNPase (*p* = 0.0005; Figure 5D–F) was higher in *Fgfr1^ind−/−^* mice. PLP (*p* = 0.0334) was increased in the cerebellum of *Fgfr1^ind−/−^* mice (Figure 5G,H). There was no effect of *FGFR* deletion on the myelin proteins CNPase (*p* = 0.955) and MBP (*p* = 0.504) (Figure 5G,H), or the myelin inhibitors SEMA3A (*p* = 0.684), TGFβ (*p* = 0.238), Lingo-1 (*p* = 0.537) in the cerebellum (Figure 5I,J).

### 2.5. FGF/FGFR Signaling Proteins and TrkB/BDNF Protein Expression in the Cerebellum of Fgfr1^ind−/−^ Mice

**Figure 6:**
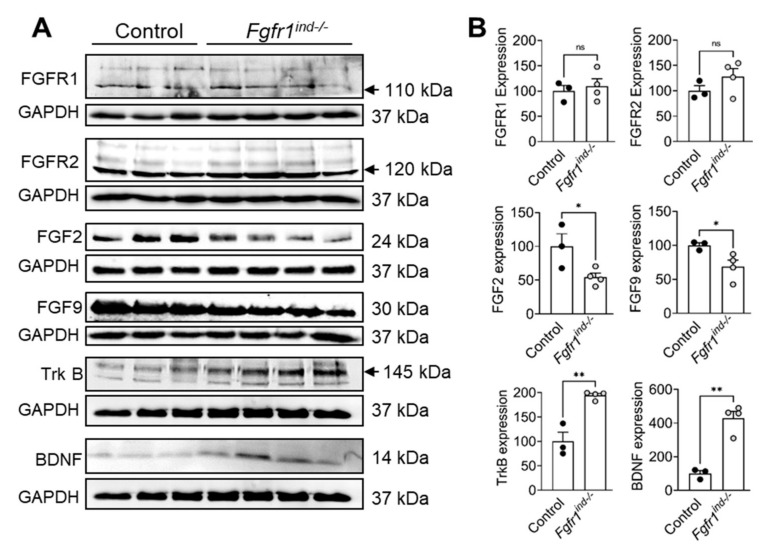
FGF/FGFR1 signaling and BDNF/TrkB expression in the cerebellum at day 62 p.i. FGFR1 and FGFR2 protein expression was not different between *Fgfr1^ind−/−^* mice and controls. The FGFR ligands FGF2 and FGF9 were decreased in *Fgfr1^ind−/−^* mice. BDNF and TrkB protein expression was increased in *Fgfr1^ind−/−^* mice (**A**,**B**). *n* = 3–4/group. Data are expressed as the mean ± SEM. ns = not significant, * *p* < 0.05, ** *p* < 0.01.

**Figure 7:**
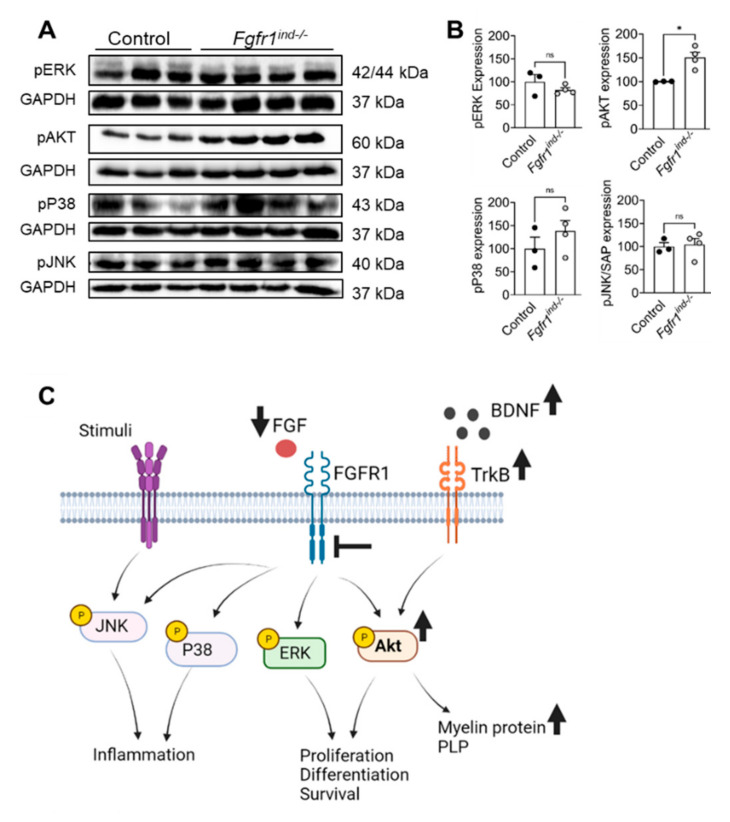
FGFR1 downstream protein expression in the cerebellum at day 62 p.i. (**A**,**B**). Phosphorylation of Akt was increased in *Fgfr1^ind−/−^* mice. ERK, P38 and JNK phosphorylation was not different between *Fgfr1^ind−/−^* mice and controls. *n* = 3–4/group. Data are expressed as the mean ± SEM. ns = not significant, * *p* < 0.05. Graphical abstract (**C**): deletion of *FGFR1* leads to increase of the myelin protein PLP via increasing phosphorylation of Akt, TrkB and BDNF expression.

In the present study, we investigated the role of oligodendroglia-specific deletion of *FGFR1* on FGF/FGFR signaling proteins and TrkB/BDNF protein expression in cerebellar lysates by western blot. *Fgfr1^ind−/−^* mice showed a decrease in the expression of FGF2 (*p* = 0.0453) and FGF9 (*p* = 0.0442) (Figure 6A,B). Phosphorylation of Akt (*p* = 0.0109, Figure 7A,B), and expression of TrkB (*p* = 0.0023) and BDNF (*p* = 0.0012) were increased in the cerebellum of *Fgfr1^ind−/−^* mice (Figure 6A,B). There was no regulation in phosphorylation of ERK1/2 in the cerebellum (Figure 7A,B).

## 3. Discussion

In the present EAE study, we investigated the function of FGFR1 in the cerebellum by generating an inducible and cell-specific deletion of *FGFR1* in oligodendrocytes. At day 62 p.i. chronic phase of EAE, myelin and axon degeneration were less severe; furthermore, lymphocyte and macrophage/microglia cell infiltration reduced. Further analyses revealed enhanced expression of the FGF/FGFR1 signaling molecule Akt activation, upregulation of BDNF/TrkB, and reduced expression of proinflammatory cytokines.

In MS and its EAE model, inflammatory infiltrates cause destruction of oligodendrocytes and myelin sheaths [37]. In EAE, oligodendrocytes directly involved in inflammation and immune modulation in the CNS [38]. FGFs (1, 2, 3, 21, 23)/FGFRs (FGFR1) play important roles in the regulation of inflammatory responses in various diseases including multiple sclerosis [39]. In the spinal cord, oligodendroglia FGFR1 and FGFR2 mice showed less infiltrating CD3(+) T-cells, B220(+) B-cells, and Mac3(+) macrophages/activated microglia during chronic phase of EAE. In agreement with recent findings in the spinal cord, the number of infiltrating CD3(+) T-cells, B220(+) B-cells, Mac3(+) macrophages/activated microglia, or IgG producing plasma cells were reduced in the cerebellum of *Fgfr1^ind−/−^* mice. Our data supports the notion that FGFR1 may directly influence the immune cell infiltration in the cerebellum.

At day 62 p.i., *Fgfr1^ind−/−^* mice showed less FGF2 and FGF9 expression in the cerebellum. To date, it is unclear why deletion of FGFRs in oligodendrocytes causes a reduction in inflammation in the spinal cord and the cerebellum [15,16]. One may speculate that deletion of FGFRs results in a reduction of FGF2 and FGF9 by regulating the downstream signaling of ERK and Akt, which may reduce inflammation by altering the proinflammatory cytokines. In the cerebellum, the proinflammatory cytokines IL1β and IL6 were reduced in *Fgfr1^ind−/−^* mice at day 62 p.i. Cytokines play a key role in recruiting activated immune cells into the CNS [40,41,42]. IL1β plays a role in neuronal degeneration via p53-mediated apoptosis of neurons [43]. IL1β-deficient mice are resistant to EAE [44], and IL1β promotes differentiation of pathogenic Th17 and lymphocyte trafficking into the CNS [45]. FGF2 was downregulated in this study. FGF2 enhances the inflammatory response of monocytes/macrophages, which increases the concentrations of cellular IL-1β in human vascular smooth muscle cells [39]. IL6-deficient animals are fully resistant to EAE [46] and astrocytic deletion of IL-6 leads less inflammatory infiltrates and decreased demyelination in the spinal cord at day 20 p.i [47]. These findings demonstrate that reduced FGF expression was associated with less inflammation and reduced proinflammatory cytokines in the cerebellum of *Fgfr1^ind−/−^* mice.

FGF2 induces downregulation of myelin proteins in vitro [48]. In contrast, conditional deletion of either *FGF2* or *FGFR* enhances remyelination in the cuprizone demyelination model at 12 weeks of cuprizone ingestion. Extended and elevated expression of FGF9 causes failure of remyelination [49]. In vitro, inhibition of FGFR in oligodendrocytes enhances myelin protein expression of PLP and CNPase [50]. Consistent with the cuprizone demyelination model and oligodendrocyte cell culture findings, our results demonstrate that oligodendrocyte-specific deletion of *FGFR1* in MOG_35-55_-induced EAE leads to increased PLP expression in the cerebellum and less FGF2 and FGF9 at day 62 p.i. Two different stains were used to assess axonal damage in the cerebellum, namely SMI-31 (NF-H) and APP. To investigate the extent of neurofilaments in the cerebellum, tissues were stained with the SMI31 antibodies. APP is a well-characterized marker to detect axonal injury in neurodegenerative diseases such as MS [51,52]. In damaged axons, the accumulation of APP occurs in case of a disturbed fast axonal transport. Analysis of axonal structure revealed that there were increased phosphorylated neurofilaments in *Fgfr1^ind−/−^* mice cerebellums. This indicates that *FGFR1* deletion in oligodendrocytes protected myelin against inflammation and resulted in reduction of axon degeneration and myelin loss in during EAE.

FGFR1 is upregulated in OPCs in active and chronic demyelinating lesions in the cortical and periventricular brain tissue of MS patients [12]. Further, extensive demyelination was present in the cerebellum of patients with progressive MS [25]. In cuprizone and lysolecithin models, FGFR1/2 double knockout mice showed less differentiated oligodendrocytes and suppressed myelin recovery [53]. In our previous EAE studies, we found oligodendrocyte-specific deletion of *FGFR1* and *FGFR2* does not alter oligodendroglial cell numbers in the spinal cord. In agreement with these recent studies, we did not observe an effect on the number of OPC or mature oligodendrocytes in the cerebellar WML at day 62 p.i. These findings suggest that the impaired *FGFR1* in oligodendrocytes does not inhibit oligodendrocyte differentiation in the cerebellum during EAE. The differences in the findings of oligodendrocyte populations could be the lack of inflammatory signals in the cuprizone and lysolecithin model in comparison to the inflammatory environment in the EAE model.

In the cerebellum, *Fgfr1^ind−/−^* mice show increased Akt phosphorylation, which is known to modulate inflammation and TrkB/BDNF expression and enhance myelination in the EAE model. In our previous studies, *Fgfr1^ind−/−^* mice had an increased expression of TrkB and increased phosphorylation of downstream Akt kinase in the spinal cord [15,16]. Akt kinase is a key downstream protein that regulates inflammation [54], and continuous activation of Akt in oligodendrocytes increases myelin synthesis [55]. Due to the suppressive character of Akt2 on the expression of Th1/Th17 cells by increasing the number of T regulatory cells [56,57], a loss of Akt3 leads to severe EAE symptoms and reduces neuroregeneration [58]. Compared to our previous study on the effects of *FGFR1* knockout in the spinal cord [16], we could not find an increased phosphorylation of ERK1/2 in the cerebellum. The importance of the functional ERK signaling for adequate remyelination and axon integrity were described in several investigations [59,60]. However, there might be an alternative pathway to ensure sufficient remyelination or decrease axonal loss without upregulation of ERK. Increased expression of BDNF and its corresponding receptor TrkB could possibly enhance remyelination [61].

## 4. Methods

### 4.1. Ethics Statement

All scientific procedures on animals were approved by the regional council of Hesse, Giessen, Germany (GI 20/23-Nr. 31/2008) in accordance with the German animal welfare act and the European legislation for the protection of animals used for scientific purposes (2010/63/EU). AVMA guidelines for the euthanasia of animals were followed. Animal studies were performed according to the guidelines of FELASA. All efforts were made to minimize pain and suffering

### 4.2. Generation of Cell-Specific FGFR1 Knockout Mice

**Figure 8:**
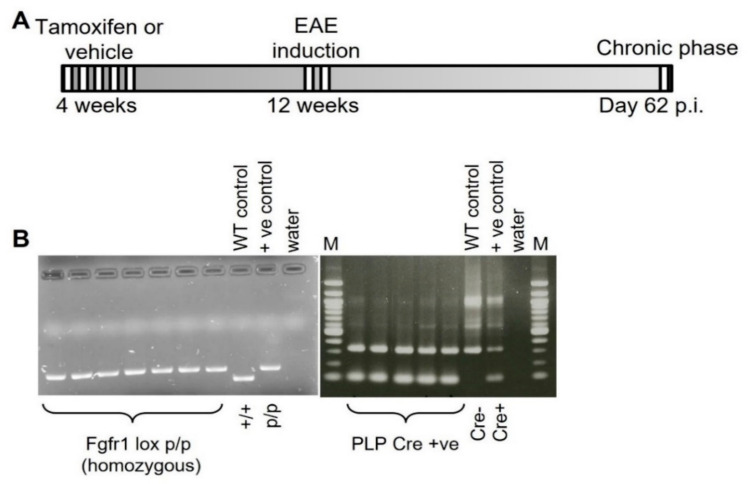
Experimental design of tamoxifen-induced conditional deletion of *Fgfr1* in oligodendrocytes and EAE induction with the MOG_35-55_ peptide. (**A**) In 4-week-old female B6.Cg-Tg(PLP1-cre/ERT)3Pop Fgfr1tm5.1Sor mice, tamoxifen was administered to induce nuclear translocation of the Cre recombinase fusion protein and subsequent deletion of floxed gene alleles. (**B**) Confirmation of FGFR1 lox and PLP-Cre expression in mutant mice by PCR and agarose gel electrophoresis. EAE was induced with the MOG_35-55_ peptide at 8–12 weeks of age, and samples were collected on day 62 p.i.

To study the cell-specific function of *FGFR1* in EAE, a conditional knockout of *FGFR1* in oligodendrocytes was generated. A detailed description of the generation of B6.Cg-Tg(PLP1-cre/ERT)3Pop:*Fgfr1^tm5.1Sor^* mice has been reported previously [16]. Briefly, B6;129S4-*Fgfr1^tm5.1Sor^*/J mice (The Jackson Laboratories, Bar Harbor, ME, USA) were crossbred with B6.Cg-Tg(Plp1-cre/ERT)3Pop/J mice (The Jackson Laboratories, Bar Harbor, ME, USA) and backcrossed to a C57BL/6J background. For induction of the Cre recombinase, 4-week-old B6.Cg-Tg(PLP1-cre/ERT)3Pop *Fgfr1^tm5.1Sor^* recipient female mice received i.p. injections of 1 mg of tamoxifen (Sigma-Aldrich, Steinheim, Germany) in a sunflower oil/ethanol mixture daily over 5 consecutive days (referred to as *Fgfr1^ind−/−^* mice). B6.Cg-Tg(PLP1-cre/ERT)3Pop *Fgfr1^tm5.1Sor^* littermate controls received a sunflower oil/ethanol mixture (referred to as controls) (Figure 8A). To confirm deletion of the *FGFR1* floxed allele and the PLP transgene, genomic DNA was isolated (DirectPCR-Tail, Peqlab, Erlangen, Germany) and mice were genotyped by PCR as recommended by The Jackson Laboratories. PCR products were detected by agarose gel electrophoresis (Peqlab, Erlangen, Germany) (Figure 8B).

### 4.3. Induction and Clinical Assessment of MOG35-55-Induced EAE

Induction and clinical evaluation of EAE was done as previously described [15,16]. Briefly, 8- to 12-week-old female *Fgfr1^ind−/−^* and control mice were injected subcutaneously in the flank with 300 µg of myelin oligodendrocyte glycoprotein peptide (MOG_35-55_; Charité Hospital, Berlin, Germany) that was emulsified in complete Freund’s adjuvant (Sigma, Steinheim, Germany) containing 10 mg of *Mycobacterium tuberculosis* (Difco, Detroit, MI, USA). Pertussis toxin was administered i.p. on days 0 and 2 postimmunization (p.i.). Symptoms were evaluated using a scale that ranged from 0 to 5 as described and presented earlier [16]. Mice were sacrificed in the chronic phase of EAE on day 62 postimmunization.

### 4.4. Histology and Immunohistochemistry

*Fgfr1^ind−/−^* mice and controls were anesthetized and transcardially perfused with 4% paraformaldehyde on day 62 p.i. The cerebellum was dissected, embedded in paraffin blocks, and stained for inflammatory infiltrates (hematoxylin and eosin) and myelin loss (Luxol fast blue/periodic acid-Schiff) as described recently [16]. For immunohistochemistry, the cerebellum was deparaffinized, rehydrated, and antigen retrieval was performed by boiling the sections in citrate buffer (10 mM, pH 6). Macrophages/activated microglia (Mac 3, clone M3/84, 1:200, Pharmingen, San Diego, CA, USA), activated B cells (B220, clone RA3-6B2, 1:200, Pharmingen, San Diego, CA, USA), T cells (CD3, clone CD3-12, 1:150, Serotec, Oxford, UK), Olig2(+), and NogoA(+) oligodendrocyte populations (Olig 2, 1:300, IBL, Gunma, Japan; NogoA(+), 1:50, Santa Cruz Biotechnology, CA, USA) were stained. To analyze the axonal damage or swelling and neurofilaments associated with axonal diameter, sections were stained with APP (1:500; Merck Millipore, Darmstadt, Germany) and SMI31 (1:500; Pharmingen, San Diego, CA, USA) antibodies. Sections were counterstained with hematoxylin, signals were detected by incubation with an avidin-biotin complex. Microscopic images were captured using Axio Scan Z1 Microscope (Carl Zeiss, Göttingen, Germany) with the ZEN software (ZEN 3.1, Carl Zeiss Microscopy GmbH, Jena, Germany). The average numbers of positive cells were normalized to an area of 1 mm^2^.

### 4.5. Protein Extraction and Western Blot Analysis

The cerebellum from *Fgfr1^ind−/−^* mice and controls were separately homogenized in an ice-cold lysis buffer with TissueRuptor (Qiagen Instruments, Hombrechtikon, Switzerland). The amount of protein was quantified (Pierce^®^ BCA Protein Assay Kit, Thermo Scientific, IL, USA) and normalized. Protein was loaded and separated by 10% SDS-PAGE and transferred (Trans Blot, Semi-dry Transfer cell, BioRad) to a nitrocellulose membrane (GE Healthcare, Amersham^TM^ Hybond ECL, Buckinghamshire, UK). The membranes were incubated overnight at 4 °C with the target-specific primary antibodies (Table 1). After that, membranes were incubated with respective secondary antibodies (Table 1) and developed with SuperSignal West Pico Chemiluminescent Substrate (Thermo, Pierce Biotechnology, Rockford, IL, USA) using an ECL ChemoCam Imager (INTAS Science Imaging Instruments GmbH, Göttingen, Germany). GAPDH (Santa Cruz Biotechnology, CA, USA) (Table 1) was used as a loading control; proteins were analyzed using the ImageJ 1.53b software (National Institute of Health, Bethesda, MD, USA).

### 4.6. Statistics

All analyses were performed blinded to the genotype. For histology and immunohistochemistry, positively labelled cells in lesions from the cerebellum were counted. Numbers of animals per group are noted in the figure legends. Student’s *t*-tests were performed to indicate differences between the genotypes. Statistical significance was set at *p* ≤ 0.05. Values are expressed as mean ± SEM. * indicates *p* < 0.05, ** indicates *p* < 0.01, *** indicates *p* < 0.001. Statistical analysis and graphs were prepared using the GraphPad Prism 9 (GraphPad Software, San Diego, CA, USA).

## 5. Conclusions

In the cerebellum, cell-specific deletion of *FGFR1* in oligodendrocytes exhibited less axonal damage, less myelin loss, and decreased inflammation in EAE. These effects in the cerebellum were associated by changes in FGF/FGFR signaling, increased BDNF/TrkB and PLP expression. Impaired FGFR1 signaling was associated with reduced expression of proinflammatory cytokines such as IL1β and IL6. In addition, reduced expression of FGF2 and FGF9 as well as increased phosphorylation of Akt were found in *FGFR1* knockout mice cerebellums. Furthermore, *FGFR1* deletion has no effect on the number of oligodendrocytes. Taken together, inhibiting FGF receptors in the CNS may protect the cerebellum against inflammation and degeneration.

## Figures and Tables

**Table 1:** List of antibodies used for Western blot and IHC.

Primary Antibodies	Host	Mol. Weight	Method	Art. No	Manufacturer
Anti-FGF2 (G2)	Mouse	18, 21, 24 kDa	WB	sc-365106	Santa Cruz Biotech, CA, USA
Anti-FGF9 (D8)	Mouse	30 kDa	WB	sc-8413	Santa Cruz Biotech, CA, USA
Anti-CNPase (B1)	Mouse	46 kDa	WB	sc-166019	Santa Cruz Biotech, CA, USA
Anti-MBP	Mouse	12, 18 kDa	WB	78896S	Cell Signaling Tech, MA, USA
Anti-PLP	Rabbit	30 kDa	WB	85971	Cell Signaling Tech, MA, USA
Anti-pERK p-44/42	Rabbit	44, 42 kDa	WB	4370s	Cell Signaling Tech, MA, USA
Anti-pAkt (Ser473)	Rabbit	60 kDa	WB	4060s	Cell Signaling Tech, MA, USA
Anti-Flg (M2F12) (FGFR1)	Rabbit	110 kDa	WB	sc-57132	Santa Cruz Biotech, CA, USA
Anti-Bek (C-8) (FGFR2)	Rabbit	120 kDa	WB	sc-6930	Santa Cruz Biotech, CA, USA
Anti-GAPDH	Mouse	37 kDa	WB	sc-365062	Santa Cruz Biotech, CA, USA
Anti-Trk B (794):sc12	Rabbit	145 kDa	WB	sc-377218	Santa Cruz Biotech, CA, USA
Anti-pro BDNF	Rabbit	14 kDa	WB	sc-65514	Santa Cruz Biotech, CA, USA
Anti-IL-1β	Mouse	17, 31 kDa	WB	12242	Cell Signaling Tech, MA, USA
Anti-IL-4	Mouse	18 kDa	WB	sc-53084	Santa Cruz Biotech, CA, USA
Anti-IL-6	Rabbit	24 kDa	WB	12912	Cell Signaling Tech, MA, USA
Anti-IL-12	Rat	70 kDa	WB	sc-65355	Santa Cruz Biotech, CA, USA
Anti-IL-17	Mouse	15 kDa	WB	sc-374218	Santa Cruz Biotech, CA, USA
Anti-TNFα	Rabbit	17, 25, 28 kDa	WB	11948	Cell Signaling Tech, MA, USA
Anti-IFNγ	Rat	16 kDa	WB	MM700	Thermo Fisher Scientific, Waltham, MA, USA
Anti-Lingo-1	Rabbit	98 kDa	WB	49389	Cell Signaling Tech, MA, USA
Anti-TGF-β	Rabbit	12, 25, 45–65 kDa	WB	3711	Cell Signaling Tech, MA, USA
Anti-SEMA3A	Rabbit	89 kDa	WB	Ab23393	Abcam, Cambridge, UK
Anti-CD200	Goat	42 kDa	WB	AF2724	R&D Systems, MN, USA
Anti-pP38	Rabbit	43 kDa	WB	9215	Cell Signaling Tech, MA, USA
Anti-pJNK	Rabbit	46, 54 kDa	WB	4668	Cell Signaling Tech, MA, USA
Mac 3 Clone M3/84	Rat	staining	IHC	553322	Pharmingen, USA
B220 clone RA3-6B2	Rat	staining	IHC	557390	Pharmingen, USA
CD3, clone CD3-12	Rat	staining	IHC	MCA 1477	Serotec, UK
MBP	Rabbit	staining	IHC	62301	Dako, Germany
Olig2	Mouse	staining	IHC	MABN50	MerckMillipore, Germany
Nogo-A	Rabbit	staining	IHC	sc-25660	Santa Cruz Biotech, CA, USA
MBP	Rabbit	staining	IHC	62301	Dako, Germany
CNPase	Mouse	staining	IHC	SMI-91R	Covance Inc., NJ, USA
APP	Mouse	staining	IHC	MAB348	Merck KGaA, Darmstadt, Germany
SMI31	Mouse	staining	IHC	SMI-31R	Covance Inc., NJ, USA
**Secondary Antibodies**	**Host**	**Method**		**Art. No**	**Manufacturer**
Anti-Rabbit-HRP	Goat	WB		7074	Cell Signaling Tech, MA, USA
Anti-mouse-HRP	Horse	WB		7076	Cell Signaling Tech, MA, USA
Mouse anti-goat	Mouse	WB		SC-2354	Santa Cruz Biotech, CA, USA
Goat anti-rat	Goat	WB		7077	Cell Signaling Tech, MA, USA

## Data Availability

The data presented in this study are available upon request to corresponding author.
